# Multicenter Study of Pelvic Nodal Autosegmentation Algorithm of Siemens Healthineers: Comparison of Male Versus Female Pelvis

**DOI:** 10.1016/j.adro.2023.101326

**Published:** 2023-07-28

**Authors:** Kareem Rayn, Garima Gokhroo, Brian Jeffers, Vibhor Gupta, Suresh Chaudhari, Ryan Clark, Anthony Magliari, Sushil Beriwal

**Affiliations:** a Department of Radiation Oncology, Columbia University Irving Medical Center, New York, New York; b Varian Medical Systems Inc, Palo Alto, California; c American Oncology Institute, Hyderabad, CA, India; d Columbia University Vagelos College of Physicians and Surgeons, New York, New York; e Division of Radiation Oncology, Allegheny Health Network Cancer Institute, Pittsburgh, Pennsylvania

## Abstract

**Purpose:**

The autosegmentation algorithm of Siemens Healthineers version VA 30 (AASH) (Siemens Healthineers, Erlangen, Germany) was trained and developed in the male pelvis, with no published data on its usability in the female pelvis. This is the first multi-institutional study to describe and evaluate an artificial intelligence algorithm for autosegmentation of the pelvic nodal region by gender.

**Methods and Materials:**

We retrospectively evaluated AASH pelvic nodal autosegmentation in both male and female patients treated at our network of institutions. The automated pelvic nodal contours generated by AASH were evaluated by 1 board-certified radiation oncologist. A 4-point scale was used for each nodal region contour: a score of 4 is clinically usable with minimal edits; a score of 3 requires minor edits (missing nodal contour region, cutting through vessels, or including bowel loops) in 3 or fewer computed tomography slices; a score of 2 requires major edits, as previously defined but in 4 or more computed tomography slices; and a score of 1 requires complete recontouring of the region. Pelvic nodal regions included the right and left side of the common iliac, external iliac, internal iliac, obturator, and midline presacral nodes. In addition, patients were graded based on their lowest nodal contour score. Statistical analysis was performed using Fisher exact tests and Yates-corrected χ^2^ tests.

**Results:**

Fifty-two female and 51 male patients were included in the study, representing a total of 468 and 447 pelvic nodal regions, respectively. Ninety-six percent and 99% of contours required minor edits at most (score of 3 or 4) for female and male patients, respectively (*P* = .004 using Fisher exact test; *P* = .007 using Yates correction). No nodal regions had a statistically significant difference in scores between female and male patients. The percentage of patients requiring no more than minor edits was 87% (45 patients) and 92% (47 patients) for female and male patients, respectively (*P* = .53 using Fisher exact test; *P* = .55 using Yates correction).

**Conclusions:**

AASH pelvic nodal autosegmentation performed very well in both male and female pelvic nodal regions, although with better male pelvic nodal autosegmentation. As autosegmentation becomes more widespread, it may be important to have equal representation from all sexes in training and validation of autosegmentation algorithms.

## Introduction

Radiation therapy (RT) is an important and effective treatment modality for many solid tumors. Contour delineation on the planning computed tomography (CT) is part of RT planning and is essential in limiting treatment toxicity while ensuring adequate target coverage.^1^^,^^2^ Unfortunately, this process is usually done manually, consuming valuable staff resources and ultimately making contouring a cost- and time-intensive task.^2^^,^^3^ Manual contouring is also associated with significant variability and inconsistency between users during the RT planning process,^4^ with some evidence suggesting that expertise can affect contour quality and subsequent patient outcomes.^5^^,^^6^ Furthermore, the contouring workload will only increase as adaptive planning becomes more common, requiring recontouring during the fractionated treatment course to account for changes of the treatment plan for parameters such as patient weight loss and tumor shrinkage.^7^

Historically, common clinical autosegmentation algorithms used were atlas-based, which required maintaining a library of self-made contours. Atlas-based methods have been shown to be feasible for contouring in patients with endometrial and cervical cancers.^8^ Previous studies on pelvis atlas-based autosegmentation generation time (before manual correction) for endometrial/cervical cancers showed ranges from 45.1 seconds for clinical target volume (CTV) contouring alone to 99.9 to 134.8 seconds for contouring of CTV plus bladder, rectum, and femoral heads.^8^^,^^9^ A recent study found that atlas-based autosegmentation of the pelvic lymph nodes (including manual adjustments) could take 12 minutes, saving time from 18.7 minutes with manual contours alone (36% decrease).^10^ In another recent study, deep learning based autosegmented contours for prostate-only radiation demonstrated a high utility for both organs at risk (OARs) and CTV, with 65% of cases requiring no more than minor edits, and a resultant median time savings of 12 minutes (30% of total time spent contouring) for physicians.^11^

There have been many studies examining the efficacy of training deep learning algorithms for autosegmentation for the pelvis.^12^, ^13^, ^14^, ^15^, ^16^, ^17^, ^18^, ^19^, ^20^, ^21^, ^22^ A recent literature review examined 74 studies on deep learning networks for autosegmentation of bladder, cervical, prostate, and rectal cancers; however, few of these studies rigorously evaluated lymph nodes.^12^ Several studies investigating the application of deep learning autosegmentation models in cervical cancer have consistently demonstrated their strong performance, which is comparable to manual contouring methods.^13^, ^14^, ^15^, ^16^ Several of these studies found that between 76% to 80% of deep learning–generated CTV contours were clinically acceptable, requiring minimal-to-no edits,^14^, ^15^, ^16^ and that 72.5% to 97% of OAR contours were clinically acceptable.^15^^,^^16^ One study found total deep learning delineation time (CTV + OARs) averaged under 15 seconds,^17^ and another study found that deep learning contouring (including manual revision) improved CTV delineation time to 9.5 minutes relative to 31 minutes with fully manual contouring.^18^ A recent study showed dose distributions for target volumes were unaffected when deep learning autosegmentation was used in cervical cancer treatment plans.^19^ Deep learning autosegmentation has been found to perform well for prostate cancer as well.^20^, ^21^, ^22^ Seventy percent of deep learning contours from a recent study were considered equal to or better than reference contours after physician review, and 95.7% of deep learning contours from the study were scored as “acceptable” or greater.^20^ In a different study that evaluated deep learning contours on a 5-point scale (1 = minimal editing needed and 5 = significant editing required), prostate-related organs scored between 1.4 and 2.8.^21^ Another study found no significant dosimetric difference in deep learning contouring of the bladder compared with manual contouring.^22^

Deep learning approaches have shown significant benefits compared with atlas-based methods in improving segmentation accuracy and efficiency.^23^ The autosegmentation algorithm of Siemens Healthineers version VA 30 (AASH; Siemens Healthineers, Erlangen, Germany) was trained and developed in the male pelvis. This work represents the first multi-institutional study to describe and evaluate an artificial intelligence (AI) algorithm for autosegmentation of the pelvic nodal region in male and female patients based on a deep image-to-image network (DI2IN).

## Methods and Materials

### Autosegmentation with a DI2IN

AASH is trained using deep learning technology that employs a DI2IN, consisting of a convolutional encoder-decoder architecture combined with a multilevel feature concatenation.^24^ An iterative process is used to ensure that during the training of the networks, the machine-generated contours become virtually indistinguishable from the human-drawn contours.^24^ The process of automatic contouring relies on a 2-step approach (Fig. E1). In the first step, the target organ region in the optimal input image is extracted using a trained deep reinforcement learning network.^25^ Deep reinforcement learning allows detection of anatomic landmarks to locate the regions of interest. The anatomic landmark detection was trained independently from segmentation algorithms with manually annotated landmark points across the human body.^25^ The result is a cropped image of the target organ, which is then used as input to create the contours in the second step based on DI2IN.^24^

### Study design

We retrospectively evaluated AASH pelvic nodal autosegmentation in both contrast and noncontrast scans (based on institutional practice) in both male and female patients treated at 5 institutions in our network. All patients used the same scanning protocol (General Electric CT scanner with 2- mm thickness). As a retrospective study, this study was institutional review board exempt. The automated pelvic nodal contours generated by AASH were evaluated by 1 board-certified radiation oncologist, specializing in prostate and gynecologic malignancies.

### Nodal contour score

A 4-point scale was used: a score of 4 is clinically usable with minimal edits; a score of 3 requires minor edits (missing nodal contour region, cutting through vessels, or including bowel loops) in 3 or fewer CT slices; a score of 2 requires major edits, as previously defined but in 4 or more CT slices; and a score of 1 requires complete recontouring of the region. The 4-point scale employed in this study has been validated in prior autosegmentation studies and has been found to be significantly associated with quantitative metrics for the commissioning process of autosegmentation.^26^^,^^27^ Pelvic nodal regions included the right and left side of the common iliac, external iliac, internal iliac, obturator, and midline presacral nodes. A separate analysis was conducted to compare the differences between contours in males and females that required minor edits at most (grouped scores 3 and 4) and those that required significant edits (grouped scores 1 and 2).

### Patient grade

In addition, patients were graded on a similar 4-point scale based on the lowest contour score of the patient's pelvic nodal regions. For example, patients who received a grade of 3 had at least 1 contour scored 3 and no contours scored 2 or lower. Patients who received a grade of 4 had scored 4 across all pelvic nodal regions, requiring no edits. Separate analysis was performed between patients who required minor edits at most (grouped grades 3 and 4) and patients who required significant edits (grouped grades 1 and 2).

### Statistical analysis

For most analyses, χ^2^ tests could not be performed, as most analyses failed to satisfy χ^2^ expected value validity requirements (less than 20% of expected values <5, no expected values <1). Instead, 2 × 4 Fisher exact tests were performed for analyses of both 4-point nodal contour scores and patient grades, using 2 online calculators.^28^^,^^29^ For analyses with grouped scores or grouped grades, 2 *P* values were calculated for each analysis, 1 using a 2 × 2 Fisher exact test, and 1 using a Yates corrected χ^2^ test. These *P* values were found using online calculators.^30^^,^^31^ A significance level of ɑ = .05 was used.

## Results

### Patient characteristics

**Figure 1:**
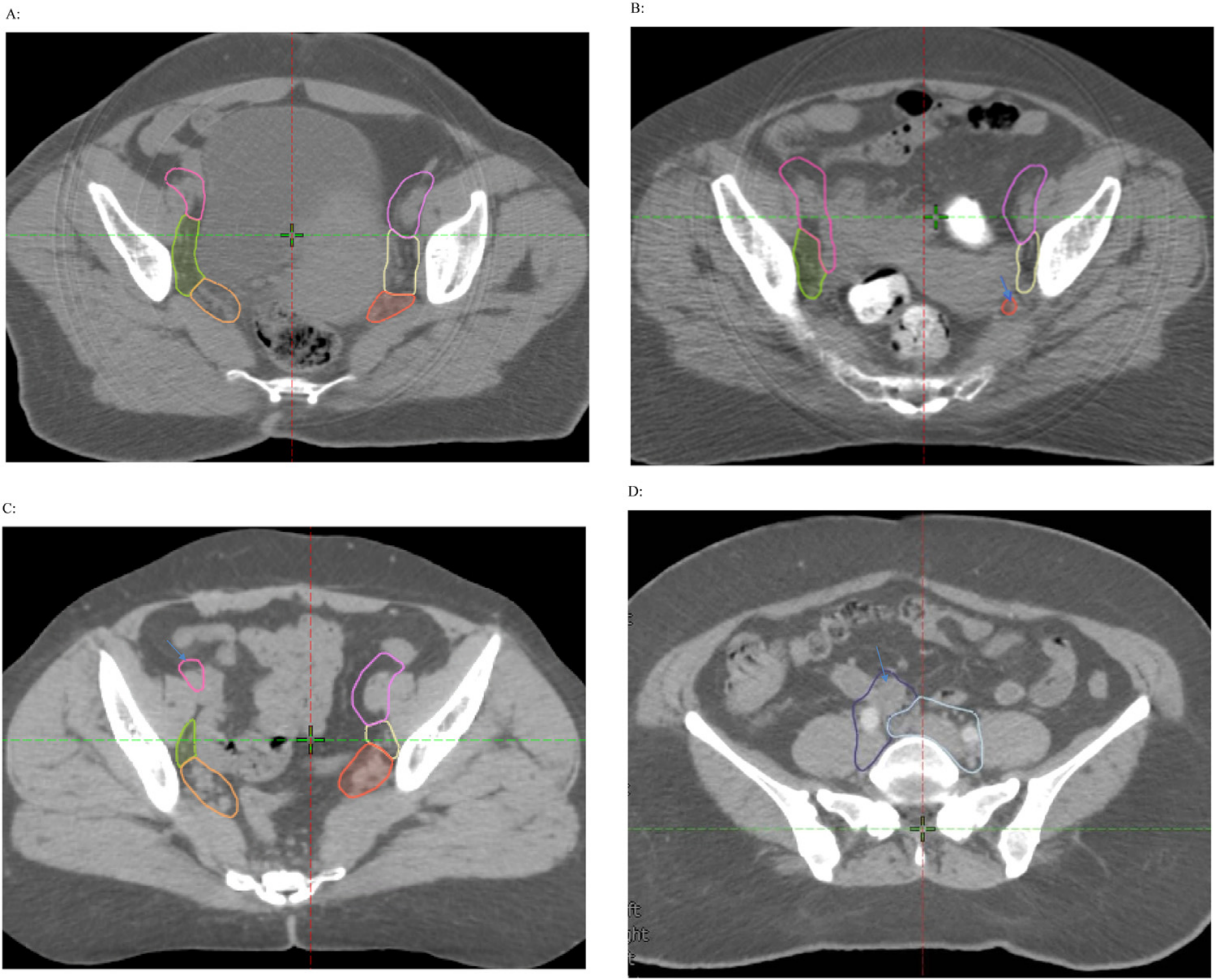
Representative axial computed tomography slices for female patients showing auto-segmented pelvic nodal regions. (A) Scored 4, requiring no edits at all. (B) Scored 2, missing internal iliac nodal region (arrow). (C) Scored 3, cutting through external iliac nodal region (arrow) in three or fewer slices. (D) Scored 3, bowel loop (arrow) included in common iliac in 2 slices.

Fifty-two female (mean age, 52.6; IQR, 45-60) and 51 male patients (mean age, 70; IQR, 67-75) were included in the study. Of the female patients, 37 had cervical cancer, 5 had endometrial cancer, 5 had rectal cancer, 2 had bladder cancer, and 3 were unknown (Table 1). Of the male patients, 35 had prostate cancer, 13 had rectal cancer, 1 had bladder cancer, 1 had penile cancer, and 1 had sarcoma (Table 1). Figure 1 and Fig. E2 show representative axial slices with autosegmented pelvic nodal regions for female and male patients, respectively.

**Table 1:** Distribution of malignancies by sex and patient grade

	Total patients	Patient grade 4	3	2	1
Distribution of malignancies in female patients					
Cervix	37	16 (43.2%)	19 (51.3%)	2 (5.4%)	0 (0%)
Endometrium	5	2 (40%)	3 (60%)	0 (0%)	0 (0%)
Rectum	5	2 (40%)	1 (20%)	2 (40%)	0 (0%)
Bladder	2	0 (0%)	0 (0%)	1 (50%)	1 (50%)
Unknown	3	1 (33.3%)	1 (33.3%)	1 (33.3%)	0 (0%)
Distribution of malignancies in male patients					
Bladder	1	0 (0%)	1 (100%)	0 (0%)	0 (0%)
Prostate	35	25 (71.4%)	7 (20%)	3 (8.6%)	0 (0%)
Penile	1	1 (100%)	0 (0%)	0 (0%)	0 (0%)
Rectal	13	9 (69.2%)	3 (23.1%)	1 (7.7%)	0 (0%)
Sarcoma	1	1 (100%)	0 (0%)	0 (0%)	0 (0%)

Total count and percentage within each cancer type are included. Patient grade = patient's lowest nodal contour score.

### Individual pelvic nodal region score distribution

Nine hundred fifteen contours were evaluated in total, with 468 and 447 pelvic nodal regions evaluated for female and male patients, respectively. The right common iliac, right internal iliac, and obturator nodal groups had a statistically significant difference between male and female patients (Table 2). When grouping contours requiring minor edits at most (grouped scores 3 and 4) together and those requiring significant edits (grouped scores 1 and 2) together, no nodal groups had a statistically significant difference in score between male and female patients (Table 3).

**Table 2:** Score distribution of each pelvic nodal region

Nodal contour score	4		3		2		1		
	F	M	F	M	F	M	F	M	*P* value
Common iliac, left (%)	43 (82.7)	48 (94.1)	7 (13.4)	3 (5.9)	2 (3.8)	0 (0)	0 (0)	0 (0)	.14
Common iliac, right (%)	39 (75)	49 (96.1)	10 (19.2)	2 (3.9)	3 (5.8)	0 (0)	0 (0)	0 (0)	.01*
External iliac, left (%)	36 (69.2)	44 (86.3)	12 (23.1)	5 (9.8)	4 (7.7)	2 (3.9)	0 (0)	0 (0)	.10
External iliac, right (%)	40 (76.9)	46 (90.2)	9 (17.3)	3 (5.9)	3 (5.7)	2 (3.9)	0 (0)	0 (0)	.15
Internal iliac, left (%)	49 (94.2)	50 (98)	2 (3.8)	1 (2.0)	1 (1.9)	0 (0)	0 (0)	0 (0)	.62
Internal iliac, right (%)	45 (86.5)	51 (100)	3 (5.8)	0 (0)	3 (5.8)	0 (0)	1 (1.9)	0 (0)	.02*
Presacral (%)	48 (92.3)	47 (95.9)	4 (7.6)	2 (4.1)	0 (0)	0 (0)	0 (0)	0 (0)	.68
Obturator, left (%)	44 (84.6)	45 (97.8)	8 (15.4)	1 (2.2)	0 (0)	0 (0)	0 (0)	0 (0)	.03*
Obturator, right (%)	42 (80.8)	45 (97.8)	9 (17.3)	1 (2.2)	1 (1.9)	0 (0)	0 (0)	0 (0)	.01*

*Abbreviations:* F = female; M = male.

⁎ Statistically significant (*P* < .05). *P* values were calculated using 2 × 4 Fisher exact test.

**Table 3:** Score distribution of each pelvic nodal region, grouped

Nodal contour score	No or minor edits (scored as 3 or 4)		Significant edits (scored as 1 or 2)			
	F	M	F	M	Fisher *P* value	Yates *P* value
Common iliac, left (%)	50 (96)	51 (100)	2 (4)	0 (0)	.50	.48
Common iliac, right (%)	49 (94)	51 (100)	3 (6)	0 (0)	.24	.25
External iliac, left (%)	48 (92)	49 (96)	4 (8)	2 (4)	.68	.69
External iliac, right (%)	49 (94)	49 (96)	3 (6)	2 (4)	1.00	1.00
Internal iliac, left (%)	51 (98)	51 (100)	1 (2)	0 (0)	1.00	1.00
Internal iliac, right (%)	48 (92)	51 (100)	4 (8)	0 (0)	.12	.13
Presacral (%)	52 (100)	49 (100)	0 (0)	0 (0)	1.00	.92
Obturator, left (%)	52 (100)	46 (100)	0 (0)	0 (0)	1.00	.92
Obturator, right (%)	51 (98)	46 (100)	1 (2)	0 (0)	1.00	.95

*Abbreviations:* F = female; M = male.

*Statistically significant (*P* < .05).

*P* values calculated with 2 × 2 Fisher exact test and Yates-corrected χ^2^ test.

### Overall pelvic nodal region score distribution

Overall, 82.5% (386) of female patient contours received a score of 4, and 95.1% (425) of male patient contours received a score of 4 (*P* < .001; Table 4). When grouping contours requiring minor edits at most together and those requiring significant edits together, 96% (450 pelvic nodal contours) and 99% (443 pelvic nodal contours) required minor edits at most for female and male patients, respectively (*P* = .004 using Fisher exact test and *P* = .007 using Yates-corrected χ^2^ test).

**Table 4:** Overall score distribution

Nodal contour score	4	3	2	1
F (%)	386 (82.5)	64 (13.7)	17 (3.6)	1 (0.2)
M (%)	425 (95.1)	18 (4.0)	4 (0.9)	0 (0)

*Abbreviations:* F = female; M = male.

*P* < .001; 2 × 4 Fisher exact test.

### Overall pelvic nodal region score distribution excluding postoperative patients

Of the 52 female patients, 10 (19.2%) included postoperative pelvis (5 cervical cancer, 4 endometrial cancer, 1 bladder cancer), whereas none of the male pelvises were postoperative. To account for the anatomic changes caused by surgery, a separate analysis comparing nonpostoperative female patients (378 total contours) to male patients was performed. For these nonpostoperative female patients, 320 contours (84.7%) received a score of 4, with a *P* < .001 compared with male patient contours (Table E1). When grouping contours requiring minor edits at most together (grouped scores 3 and 4) and those requiring significant edits together (grouped scores 1 and 2), 97% (365 pelvic nodal contours) required no or minor edits for female patients (*P* = .01 using Fisher exact test and *P* = .02 using Yates-corrected χ^2^d test).

### Patient-level analysis of overall pelvic nodal region score distribution (patient grade)

Of the 52 total female patients, 21 (40.4%) received a grade of 4, requiring no edits, and 24 (46.2%) received a grade of 3 (Table 5). Compared with female patients, of the 51 total male patients 36 (70.6%) received a grade of 4 and 11 (21.6%) received a grade of 3 (*P* = .009; Table 5). The percentage of patients requiring minor edits at most (grouped grades 3 or 4) was 87% (45 patients) and 92% (47 patients) for female and male patients, respectively (*P* = .53 with Fisher exact test and *P* = .55 with Yates-corrected χ^2^ test).

**Table 5:** Overall patient grade distribution

Patient grade	4	3	2	1
F (%)	21 (40.4)	24 (46.2)	6 (11.5)	1 (1.9)
M (%)	36 (70.6)	11 (21.6)	4 (7.8)	0 (0)

*Abbreviations:* F = female; M = male.

Patient grade = patient's lowest nodal contour score. *P* = .009; 2 × 4 Fisher exact test.

## Discussion

The use of AI in clinical practice is no longer a distant idea, with 45% of surveyed professionals already using AI-based autocontouring tools in a recent study.^32^ This usage extends beyond OAR contouring to include prostate, thorax, and bladder tumor contours. This trend is confirmed by a study of European medical physicists, where 37% reported using AI in their work, mainly for contouring and treatment planning.^32^ With this widespread adoption, there is an urgent need for clear guidelines and training on the safe and effective use of AI, its limitations, and how to avoid potential issues that our study helps to address. Previous studies have shown the effectiveness of deep learning for automated elective lymph node segmentation for head and neck cancer RT^33^ and atlas-based autosegmentation for postoperative RT planning in endometrial and cervical cancers.^8^ However, this is the first multi-institutional study to describe and evaluate an AI algorithm for autosegmentation of the pelvic nodal region based on a DI2IN. These results demonstrated the model is clinically usable in both male and female pelvic nodal regions, with 99% and 96% of contours requiring no or minor edits, respectively. It is notable that these results were obtained in predominantly south Asian patients, despite the model being primarily trained on Caucasian males. However, AASH did perform better in male patients in comparison to female patients, with a statistically significant difference in overall contour score and overall patient grades. There were no statistically significant differences between male and female patients for any individual nodal groups. A separate analysis, which excluded postoperative female contours to assess if the results would be similar to male contours, revealed no statistical difference in outcome.

Overall, 17.5% of generated female contours required at least a minor edit, of which 3.8% required major changes, and 4.9% of generated male contours required at least a minor edit, of which 0.9% required major changes. Common revisions that needed to be made for contours requiring edits included missing segments of the iliac nodal group, missing obturator node completely on 1 side, including 1 or more loops of bowel in the iliac nodal contours, missing a few slices of a presacral nodal group, and extending the external iliac contour too far anteriorly. These revisions were most often required in cases of aberrant vessels (Fig. E2C). Because it is unlikely that training for the deep learning algorithm included every nodal aberration of the nodal anatomy, it is unsurprising that performance changes are observed in patients with aberrant vascular anatomy.

Contouring variation is common among radiation oncologists, which can affect patient treatment plan quality and outcomes^1^^,^^34^ and may even result in increased toxicities and decreased survival.^35^^,^^36^ This variation in contour delineation has been studied extensively in the context of clinical trials, where deviations from protocols are recorded in the quality assurance process. A recent review found that significant deviations in target definition occurred in as much as 13% of RT plans across 5 different trials.^32^ The improved consistency offered by autosegmentation can result in increased plan quality, for example, through an enhanced ability to assess plan quality using tools such as clinical goals and by reducing inter-institution variations.^32^^,^^37^

Even though all evaluators subjectively reported significant time saving and have adopted AASH in their practice, a quantitative assessment of time saving was not conducted. Several studies have shown time saving associated with automatically generated contours compared with manually generated contours.^38^^,^^39^ Specifically, 1 study estimated time savings are expected when changing less than ∼40% of a generated contour.^39^ Given that the AASH resulted in greater than 95% of contours accepted by treating physicians with no or minor edits for both sexes, the time-saving potential is substantial with AASH. Another limitation is that contours were assessed by an expert radiation oncologist in prostate and gynecologic cancer, potentially limiting generalizability of the results. This limitation was discussed in the American Association of Physicists in Medicine Task Group Report 273 recommendations on machine learning: the results of a clinical reader performance assessment, such as in our study, may not be generalizable to all clinical settings because of the variability of the study patients and of the clinical evaluator.^40^ However, because this study's assessing physician was involved in developing the guidelines for pelvic nodal segmentation, the ratings may actually be more stringent, reflective of a strict interpretation of published guidelines. The study was further limited by the small number of counts when broken down into nodal groups, which necessitated accommodation during statistical analysis. Further studies can ensure a larger sample size, ensuring adequate counts for each of the nodal groups. American Association of Physicists in Medicine Task Group Report 273 also notes that a large, population-representative sample size is crucial during training and validation for generalizability and accurate evaluation of machine learning performance.^40^ Although AASH pelvic nodal autosegmentation performed very well in both male and female pelvic nodal regions, AASH performed better on male patients overall compared with female patients (*P* = .009). This highlights both the importance of training algorithms on a data set representative of the target population (eg, both male and female pelvises) and the importance of training said algorithm with a sufficient sample size. Another limitation of the study is the lack of quantification of dosimetric effect of AASH compared with manually generated contours. Dosimetric effect of AASH can be evaluated in further studies.

## Conclusion

The AASH pelvic nodal autosegmentation algorithm performed well and was usable for both male and female pelvic nodal regions. It likely performed better in the male pelvis because it was trained and validated in the male pelvis. To ensure equal representation, it is important to include data from both sexes in the training and validation of future autosegmentation algorithms.

## Disclosures

Kareem Rayn reports an ASTRO-Varian funded fellowship. Garima Gokhroo, Vibhor Gupta, and Suresh Chaudhari report being employed by a Varian-funded entity. Ryan Clark, Anthony Magliari, and Sushil Beriwal report being employed by Varian.

## Appendix Supplementary materials

Supplementary material Image, application 1

## Footnotes

Supplementary material associated with this article can be found in the online version at doi:10.1016/j.adro.2023.101326.
