# Supplementary material for: Multicenter Study of Pelvic Nodal Autosegmentation Algorithm of Siemens Healthineers: Comparison of Male Versus Female Pelvis
Source: Adv Radiat Oncol. 2023 Jul 28;9(2):101326. doi: 10.1016/j.adro.2023.101326 (PMC10885554; doi:10.1016/j.adro.2023.101326)
Supplement: Supplementary material [file mmc1.docx]

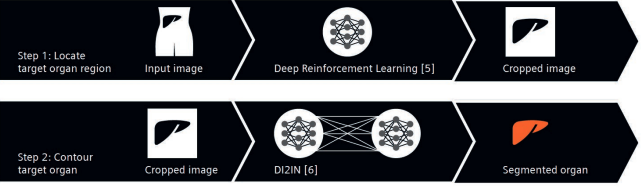


Supplementary Figure 1: Two step algorithm for deep learning based contouring

Supplementary Figure 2A:


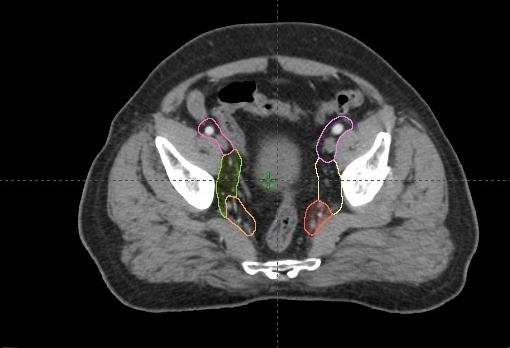


Supplementary Figure 2B:


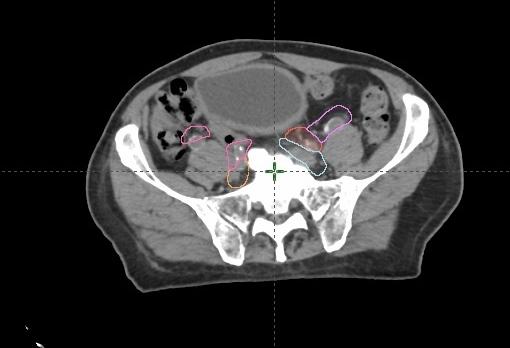


Supplementary Figure 2C:
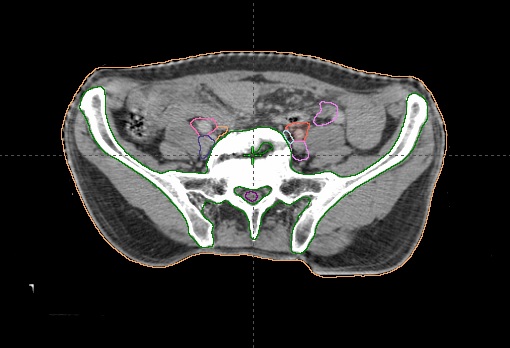


Supplementary Figure 2D:
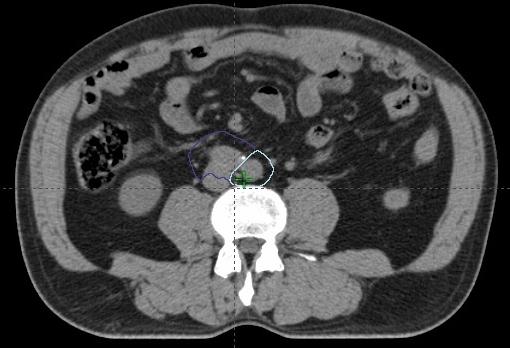


Supplementary Figure 2: Representative axial CT slices for male patients showing auto-segmented pelvic nodal regions. (A) Scored 4, requiring no edits at all. (B and C) Scored 2, missing or cutting through external iliac region in more than 3 slices (arrows). Note the aberrant external iliac vessels in 2C. (D) Scored 2, missing common iliac nodes in more than 3 slices.

| Nodal contour score: | 4 | 3 | 2 | 1 |  |
| --- | --- | --- | --- | --- | --- |
| F(excluding post-operative), (%) | 320(84.7) | 45(11.9) | 13(3.4) | 0(0) |  |
| M, (%) | 425(95.1) | 18(4.0) | 4(0.9) | 0(0) |  |
| Supplementary Table 1: Overall score distribution, excluding female postoperative patients. F=female, M=male. P<0.001 (2x4 Fisher’s exact test). | | | | | |
